# Supplementary material for: A Comprehensive Assessment of Ultraviolet-Radiation-Induced Mutations in Flammulina filiformis Using Whole-Genome Resequencing
Source: J Fungi (Basel). 2024 Mar 20;10(3):228. doi: 10.3390/jof10030228 (PMC10971301; doi:10.3390/jof10030228)
Supplement: Supplementary file 1 [file jof-10-00228-s001.zip › Supplementary Material S8/KEGG annotation/out/64381550635650.os/KO/out_map/map04744.html]

KEGG PATHWAY: Phototransduction - Reference pathway


|  |  |
| --- | --- |
| **Phototransduction - Reference pathway** |  |

[
Pathway menu
| Organism menu
| Pathway entry
| Show description
| User data mapping
]

Phototransduction is a biochemical process by which the photoreceptor cells generate electrical signals in response to captured photons. The vertebrate cascade starts with the absorption of photons by the photoreceptive pigments, the rhodopsins, which consist of a membrane embedded chromophore, 11-cis-retinal, and a G-protein-coupled receptor, opsin. The photon isomerizes 11-cis-retinal to all-trans-retinal which induces a structural change that activates the opsin. This triggers hydrolysis of cGMP by activating a transducinphosphodiesterase 6 (PDE6) cascade, which results in closure of the cGMP-gated cation channels (CNG) in the plasma membrane and membrane hyperpolarization. The hyperpolarization of the membrane potential of the photoreceptor cell modulates the release of neurotransmitters to downstream cells. Recovery from light involves the deactivation of the light- activated intermediates: photolyzed rhodopsin is phosphorylated by rhodopsin kinase (RK) and subsequently capped off by arrestin; GTP-binding transducin alpha subunit deactivates through a process that is stimulated by RGS9.

|  |  |
| --- | --- |
| Reference pathway | 100% |
